# A dengue virus infection in Ethiopia: a systematic review and meta-analysis

**DOI:** 10.1186/s12879-024-09142-1

**Published:** 2024-03-06

**Authors:** Eshetu Nigussie, Daniel Atlaw, Getahun Negash, Habtamu Gezahegn, Girma Baressa, Alelign Tasew, Demisu Zembaba

**Affiliations:** 1 Department of Medical Laboratory Science, School of Medicine, Madda Walabu University, Addis Ababa, Ethiopia; 2 Department of Biomedical Science, School of Medicine, Madda Walabu University, Addis Ababa, Ethiopia; 3 Department of Public Health, School of Health Science, Madda Walabu University, Addis Ababa, Ethiopia

**Keywords:** Dengue, Dengue virus, Systematic review, Meta-analysis, Ethiopia

## Abstract

**Background:**

Dengue is caused by a positive-stranded RNA virus called dengue virus, which is spread by Aedes mosquito species. It is a fast-growing acute febrile disease with potentially lethal consequences that is a global public health problem, mostly in tropical and subtropical countries. In Ethiopia, dengue fever is understudied, although the virus is still being transmitted and viral infection rates are rising. This systematic review and meta-analysis was aimed at estimating the pooled prevalence of DENV infection in Ethiopia.

**Methods:**

A literature search was done on the PubMed, Hinari and Google Scholar databases to identify studies published before July, 2023. Random effects and fixed effects models were used to estimate the pooled prevalence of all three markers. The Inconsistency Index was used to assess the level of heterogeneity.

**Results:**

A total of 11 studies conducted on suspected individuals with dengue fever and acutely febrile participants were included in this review. The majority of the studies had a moderate risk of bias and no study had a high risk of bias. A meta-analysis estimated a pooled IgG prevalence of 21% (95% CI: 19–23), a pooled IgM prevalence of 9% (95%CI: 4–13) and a pooled DENV-RNA prevalence of 48% (95% CI: 33–62). There is evidence of possible publication bias in IgG but not in the rest of the markers.

**Conclusion:**

Dengue is prevalent among the dengue fever suspected and febrile population in Ethiopia. Healthcare providers, researchers and policymakers should give more attention to dengue fever.

**Supplementary Information:**

The online version contains supplementary material available at 10.1186/s12879-024-09142-1.

## Introduction

Dengue virus (DENV) is a positive-stranded RNA virus of the family Flaviviridae and genus Flavivirus that is spread by arthropods. The four different dengue virus serotypes (DENV 1–4) are all spread by Aedes mosquitoes, primarily *Aedes aegypti* and to a lesser extent, *Aedes albopictus* [1, 2]. All serotypes are known to produce the whole spectrum of dengue fever (DF) illnesses and share similar geographic patterns and host/vector interactions [3, 4].

Dengue fever is a fast-growing acute febrile disease with potentially lethal consequences that is a global public health problem, mostly in tropical and subtropical countries [5, 6]. This infection can result in a variety of clinical illnesses, from asymptomatic or moderate febrile disease to classic DF to the most serious types of illness, dengue hemorrhagic fever (DHF) and dengue shock syndrome (DSS) [7, 8]. The main clinical signs of each category would be a persistent high fever lasting 2–7 days, bleeding indicated by petechiae, epistaxis, a positive tourniquet test, or thrombocytopenia, and shocks from plasma leakage indicated by hemoconcentration (hematocrit above 20%), pleural effusion, and ascites [9].

Dengue fever is endemic in many countries across the World Health Organization (WHO) regions of Africa, the Americas, the Eastern Mediterranean, Asia, Australia, and the Western Pacific [4, 5]. While the Americas, South-East Asia, and the Western Pacific are the most severely affected, Asia accounts for 70% of the global DF disease burden (10). Dengue virus infects 390 million individuals worldwide, with 96 million developing clinical symptoms that result in 500,000 hospitalizations and 25,000 fatalities each year [5, 10].

The epidemiology of DF in Africa is poorly understood, even though all DENV serotypes circulate in many of the continent’s nations, and the vector mosquitoes are abundant in the neighboring Middle East and Sub-Saharan Africa [9]. With an estimated burden of 25% (21–29%) by immunoglobulin G (IgG), 10% (9–11%) by immunoglobulin M (IgM), and 14% (12–16%) by viral ribonucleic acid (RNA) assays, DENV infection appears to be a considerable burden for public health in the region of Sub-Saharan Africa [11]. Additionally, numerous countries in the region have suffered DF outbreaks that have been reported to the Africa Center for Disease Control and Prevention (CDC) [12], including Burkina Faso in 2016 and 2017, Côte d’Ivoire in 2017, Cape Verde in 2009, and Egypt in 2015.

Ethiopia has a low level of research on DF despite the fact that the virus is still being transmitted and viral infection rates are rising [13]. In the first DF outbreak reported in 2013, which was in the Dire Dawa City administration in the east of the country, 12,000 suspected cases of DF were registered. Of these, 88 cases were confirmed by enzyme-linked immunosorbent assay (ELISA) and real-time polymerase chain reaction (RT-PCR), and 50 of these cases were found to be positive for DENV infection [14]. In Dire Dawa City, Godey Town, and the Adar area of the Afar region the next year, numerous further outbreaks were noted year-round fashion [15, 16]. In a few serological studies, DENV infections from various regions of the country were reported, including 7.5% current and 13.0% past DENV infections from northwest Ethiopia [7], 22.9% anti-IgG and 7.9% anti-IgM DENV infections from the Borena zone in southern Ethiopia [17], and 25.1% anti-IgG and 8.1% anti-IgM DENV infections from the Arba Minch district [8]. Overall, the prevalence of DENV infection is varies in Ethiopia [18–23].

The burden of dengue is increasing globally. The main contributors to this global spread are increased human movement in arbovirus endemic areas, a lack of vector control, urbanization, and deforestation. Understanding cross-reactivity patterns is also important because cross-reactive pre-existing heterotypic arbovirus antibodies can potentially enhance infection of a heterologous virus strain via antibody-dependent enhancement [24, 25].

Due to its global distribution, DENV infections should be closely monitored and any outbreaks should be quickly identified to reduce the resulting mortality and morbidity. However, it is well known that low-income countries like Ethiopia lack the infrastructure and resources required for effective surveillance of this disease. This systematic review and meta-analysis aimed at estimating the pooled prevalence of DENV infection markers; DENV-specific IgG, IgM, and DENV RNA to compile available evidences in Ethiopia.

## Methods

This systematic review and meta-analysis was conducted by the Preferred Reporting Items for Systematic Reviews and Meta-Analyses (PRISMA) guidelines [26]. The review protocol was registered on Prospero (CRD42023445570).

### Eligibility criteria

This review was considered studies that have reported the prevalence of dengue virus infection in Ethiopia. The study participants were of both sexes and any age range. We considered observational studies (cross-sectional and Case-control) in both outbreaks and out-of-outbreak periods. Studies that reported a prevalence of at least one of the three biological markers of DENV-RNA, IgM, or IgG were included. We considered all studies written in English language and published before July, 2023. Review studies, studies with only abstracts, studies with incomplete data, studies report infection rates in non-human animals, and entomologic and vector-related studies were excluded.

Dengue infection was defined as febrile illness presenting with fever and at least two of the following clinical manifestations: headache, retro-orbital pain, myalgia, arthralgia, rash, hemorrhagic manifestations, and leukopenia confirmed by laboratory criteria through the detection of DENV RNA RT-PCR or NS1 antigens using ELISA and/or rapid tests [27].

### Search strategy

To find pertinent literature published on DENV infection in Ethiopia, a comprehensive search of PubMed, Hinari, and Google Scholar was carried out. The databases were queried search by using the keywords “Dengue”, “Dengue virus”, “Dengue infection”, “Dengue fever” and “DENV” in combination with the Ethiopia context. A manual search that consists of scanning reference lists of eligible studies and relevant reviews was performed. The search strategy used in PubMed and Hinari is demonstrated in supplementary file 1.

### Study selection

The titles and abstracts of all retrieved citations were imported into the EndNote reference manager. Duplicates were removed using this software. Two investigators independently screened titles and abstracts for eligibility based on predetermined selection criteria. The full texts of articles thought to be potentially eligible were retrieved for further assessment. Further, the investigators independently assessed the full text of each study for eligibility, and consensually retained studies to be included. Any discrepancies that arise between the reviewers at each level of the selection process were solved through conversation.

### Data extraction

The data have been extracted using a predefined, piloted and standardized data extraction form. Two investigators independently extracted data, including the name of first the author, year of publication, study design, sample size, study setting (clinical or community), target population (suspected, acute febrile patients), time frame (during outbreak or out of outbreak periods), types of diagnostic techniques (RT-PCR, ELISA and (immunofluorescent assay (IFA)), and types of detected biological markers (RNA, IgM and IgG).

### Quality assessment

Two reviewers assessed the quality and risk of bias of all articles included in this review using a modified version of the Critical Appraisal Tool for prevalence studies designed by the Joanna Briggs Institute (JBI) [28]. The risk of selection bias, confounding bias, and bias relating to measurement and data analysis were all assessed using the JBI tool. Each question was answered either with “yes,” “no,” “unclear” or “not applicable”. The number of questions for each study that had a “yes” response was used to determine a score. According to this score, studies were categorized into three groups based on their risk of bias: high risk (a score of 0–3), intermediate risk (a score of 4–6), and low risk (a score of 7–9).

### Statistical analysis

To determine the pooled prevalence of DENV markers, the retrieved data were analyzed using Stata software (version 17; StataCorp LLC, Texas, USA). Multiple markers were provided by some researches and the estimates were input independently in the meta-analysis. In order to quantify the effect, we used the prevalence estimates from each study. The stated prevalence estimates and the sample size of each study were used to compute the standard error (SE). A fixed-effects model (for DENV-IgG) and random-effects model (for DENV-IgM and RNA) were used to generate summary pooled prevalence data with 95% confidence intervals (CI) and the results present in forest plots. Heterogeneity was assessed using the Inconsistency Index (I^2^). To check for potential publication bias, funnel plots were created and visually examined. Egger’s tests were used to confirm presence of heterogeneity.

## Results

### Study selection and characteristics

**Fig. 1:**
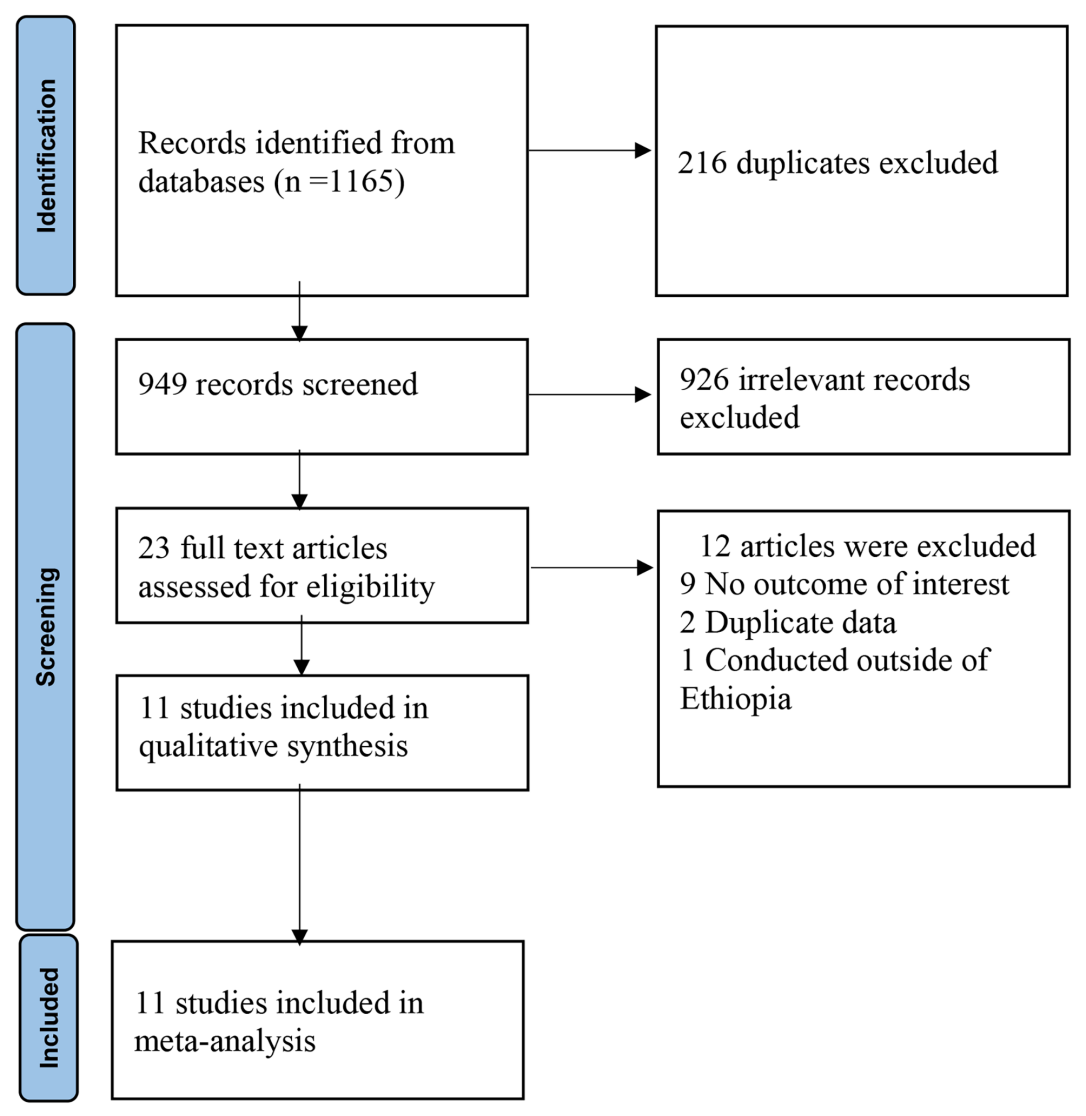
PRISMA flow diagram illustrating studies selection process

A total of 1,165 records were retrieved from database searches. After the removal of duplicates and screening, 11 studies were finally included in the review (Fig. 1). The methodological quality of studies ranged from intermediate to low risk of bias. Four (36.4%) studies had a low, 7(63.6%) had a moderate risk and no study had a high risk of bias. The majority of the included studies were cross-sectional and two were case-control studies. Six studies were conducted on suspected dengue fever participants whereas five were among acute febrile participants (Table 1).

**Table 1:** The characteristics of articles included in the systematic review and meta-analysis by time frame, diagnostic assay, design, and population

S/N	Authors	Study Design	Study setting	Population	Sample size	Time frame	Diagnostic assay	Prevalence		
								RNA	IgM	IgG
1	Ahmed et al. (2016)	Cross-sectional	Clinical	Suspected	57	During outbreak	RT-PCR	0.579		
2	Akelew et al. (2022)	Cross-sectional	Clinical	Acute febrile	200	Out of outbreak	ELISA		0.08	0.13
3	Eshetu et al. (2020)	Cross-sectional	Clinical	Acute febrile	529	Out of outbreak	IFA		0.08	0.251
4	Ferede et al. (2018)	Cross-sectional	Clinical	Acute febrile	600	Out of outbreak	ELISA		0.19	0.21
5	Geleta EN (2019)	Cross-sectional	Clinical	Acute febrile	519	Out of outbreak	IFA		0.08	0.229
6	Gutu et al. (2021)	Case-control	Clinical	Suspected	21	During outbreak	RT-PCR	0.72		
7	Mekuriaw et al. (2022)	Cross-sectional	Clinical	Suspected	12	During outbreak	RT-PCR	0.5		
8	Mesfin et al. (2022)	Case control	Clinical	Suspected	20	During outbreak	RT-PCR	0.3		
9	Shimelis et al. (2023)	Cross sectional	Clinical	Acute febrile	407	Out of outbreak	ELISA		0.02	
10	Sisay et al. (2022)	Cross sectional	Clinical	Suspected	60	During outbreak	RT-PCR	0.22		
11	Woyessa et al. (2014)	Cross sectional	Clinical	Suspected	88	During outbreak	RT-PCR	0.568		

### Prevalence of dengue virus infection

**Fig. 2:**
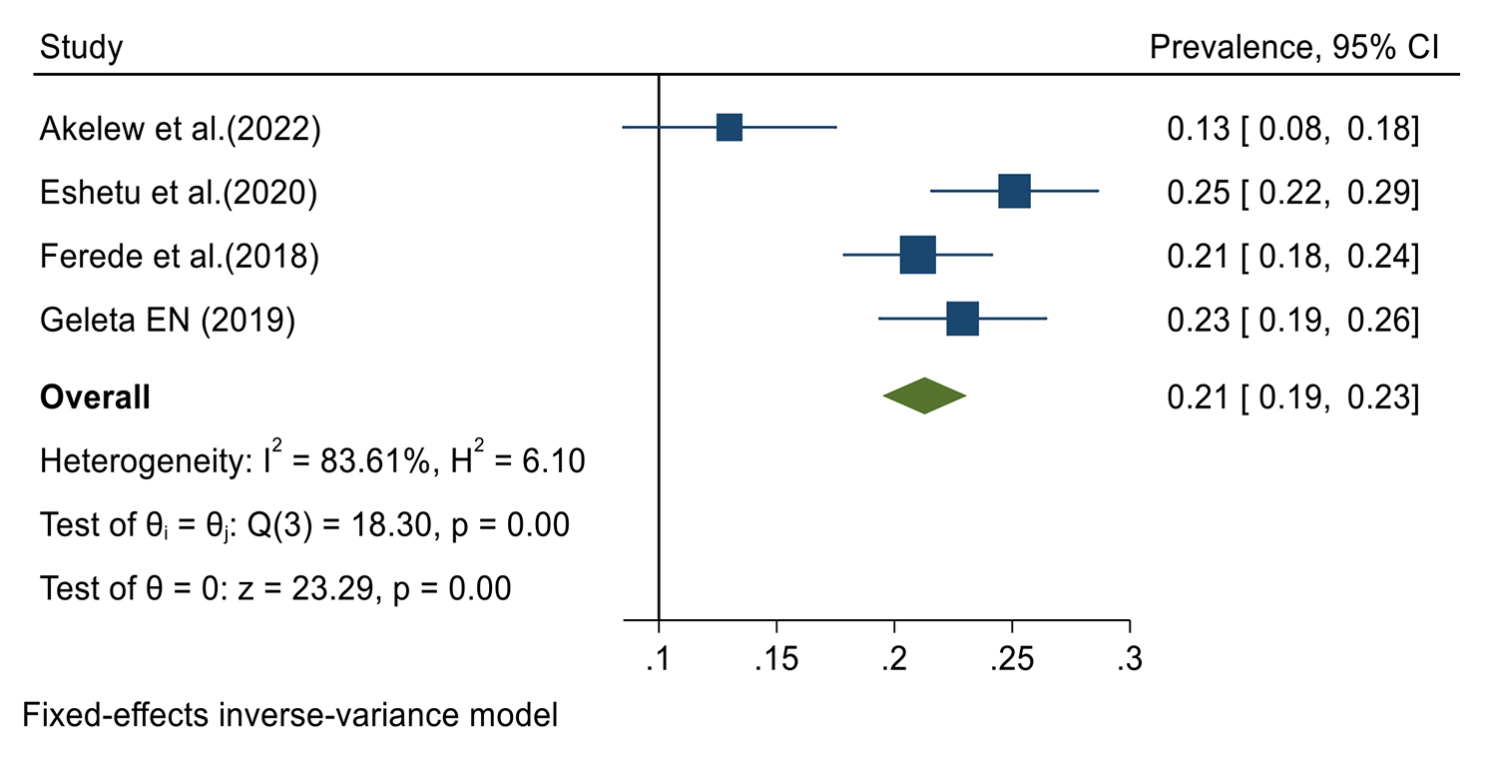
Meta-analysis of dengue virus immunoglobulin G prevalence in Ethiopia

**Fig. 3:**
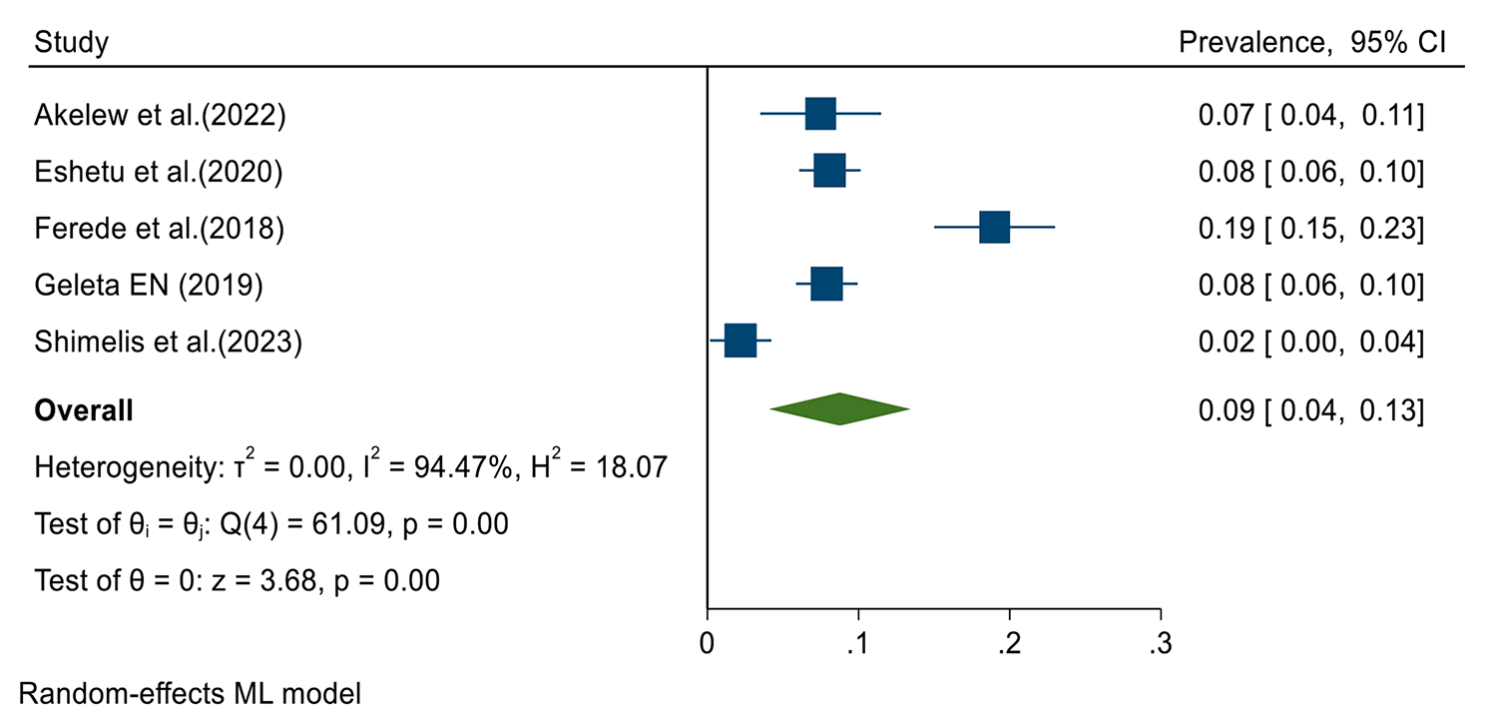
Meta-analysis of dengue virus immunoglobulin M prevalence in Ethiopia

**Fig. 4:**
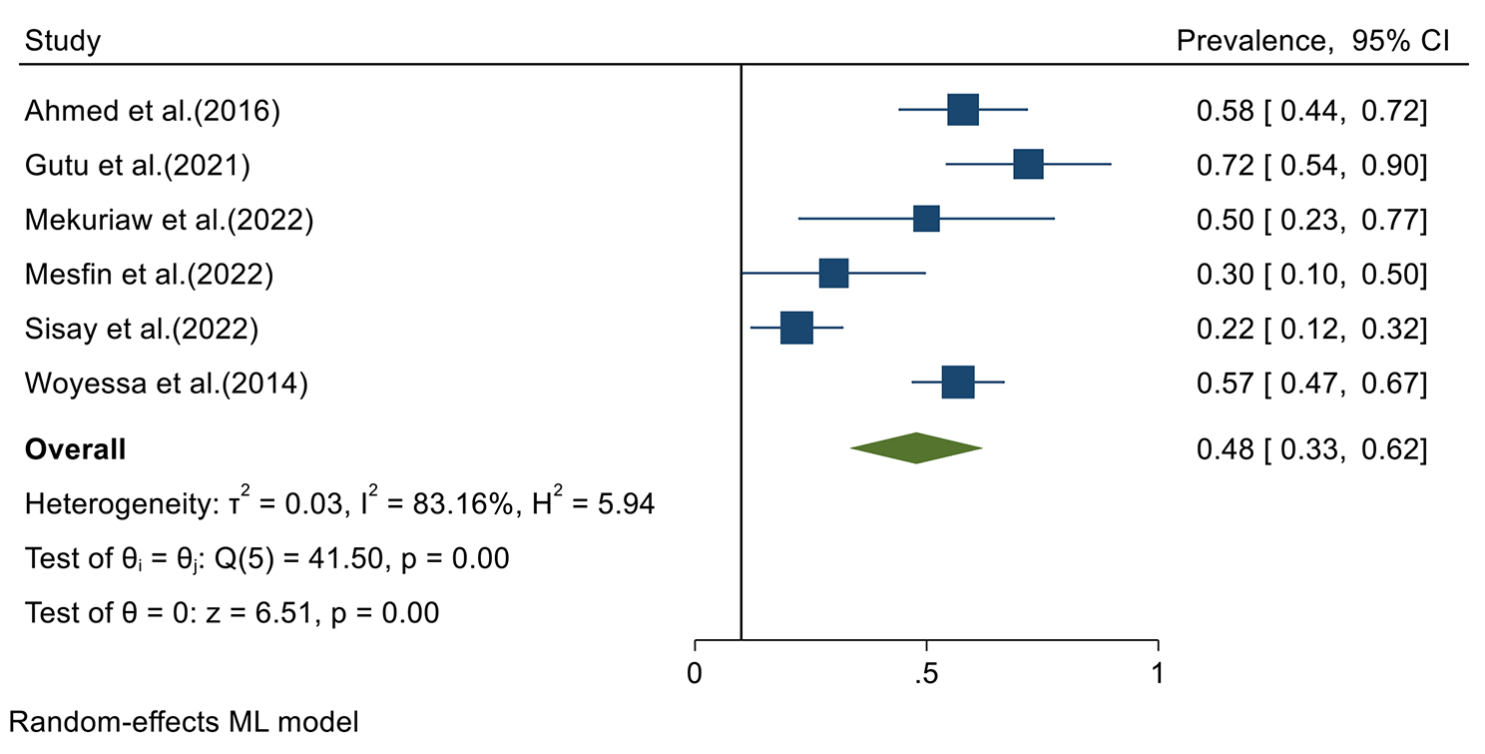
Meta-analysis of dengue virus RNA prevalence in Ethiopia

Data from all included studies were analyzed in the quantitative meta-analysis to estimate the pooled prevalence. Meta-analysis was performed separately for each of the DENV infection markers (IgG, IgM and RNA). Fixed effects analysis estimated a pooled prevalence of IgG 21% (95% CI: 19–23) (Fig. 2). Random effects analysis estimated a pooled prevalence IgM of 9% (95%CI: 4–13) and a pooled DENV-RNA prevalence of 48% (95% CI: 33–62) (Figs. 3 and 4). Significant levels of heterogeneity between studies were found in all three meta-analysis: 83.61%, 94.74%, and 83.16% for IgG, IgM and RNA respectively (Figs. 2, 3 and 4). Visual inspection of the generated funnel plots revealed evidence of potential publication bias in all three meta-analyses (Supplementary Files 2–4). But, the egger’s regression test showed that there is publication bias in DENV IgG studies (*p* = 0.002), but no publication bias was detected among DENV-RNA (*p* = 0.7) and IgM (*p* = 0.052) markers.

## Discussions

This systematic review and meta-analysis of the prevalence of DENV infection among dengue fever suspected individuals and acute febrile patients in Ethiopia showed increasing prevalence estimates with a meaningfully high level of heterogeneity that differs according to the infection marker of interest.

Meta-analysis results have shown that the prevalence of dengue virus in Ethiopia is 9%, 21%, and 48% for DENV-IgM, IgG, and DENV-RNA respectively. According to the finding of this review, 9% of the Ethiopian population has developed acute DENV infection and 21% have had a history of exposure to DENV at some point in their life. The seroprevalence of DENV-RNA was significantly higher than that of DENV-IgM and IgG; this might be most studies that determined DENV-RNA were during outbreak periods. The estimated IgM prevalence is lower compared to IgG and RNA; RNA is detectable in patients only from infection up to six days after the onset of the fever. Immunoglobulin M is detectable five days after the onset of fever up to three months, while IgG appear on the tenth day after the onset of fever and can continue for many years [29].

The prevalence of DENV-IgG was in agreement with results reported from Sub-Saharan Africa [11], and Africa [30]. In contrast, the findings of this review are lower than results reported from Sudan [31]. An estimated IgM prevalence is similar to reviews reported from Africa [30] and Sub-Saharan Africa [11] and lower than report from Sudan [31]. In addition DENV-RNA finding of this review is higher than reviews reported elsewhere [11, 30]. This discrepancy might be due to the selection and time frame of the included studies. For instance, studies conducted during ongoing epidemics or following outbreaks are likely to contribute to a higher number of dengue-positive cases [32]. Overall, the significant difference in prevalence estimate might be due to sample size, geographical variation, time frame of sample collection, type of assay methods, and way of life of the community [33].

We found that the pooled prevalence estimate of all DENV markers is alarming in Ethiopia. The DENV-specific RNA can record a four to five-fold increase during outbreak. This evidence indicates that there is a circulation of the virus in this country, as a result the residents are at high risk of sever dengue infections such as DHF and DSS.

The prevalence outcome of this review may be an indication of the ineffectiveness of current public health efforts to control DENV vector transmission. Weak health systems (like inadequate laboratory capacity to differentiate dengue from other febrile illnesses, such as malaria, yellow fever, typhoid fever, and leptospirosis, that share a similar clinical presentation) in low-income settings can be attributed to the failure to sustain effective and ongoing interventions to manage vector-borne disease [34–36]. The growing speed of urbanization with poor infrastructure increases mosquito breeding sites. The ecological environment and climatic patterns of tropical and subtropical regions where countries like Ethiopia located favor vector long-life and egg conservation and development [37–41]. Additionally, the appearance of insecticide resistance in DENV vectors and human mobility in the country as well as in the region increase the challenge of controlling the spread of those vectors [38]. The majority of studies included in this review were reported from the eastern part of the country as well as the south and northwest of Ethiopia. This may suggest that there is dengue virus circulation in Ethiopian neighboring countries including Sudan, Kenya and Djibouti, which raises the possibility of cross-border transmission, particularly with the open borders and free mobility between these countries.

The findings of this review contains important information for researchers, health policymakers and clinical practitioners. Such studies will generate base-line information for the development of effective public health policies for dengue fever prevention and control. A surveillance program is also required to study the actual prevalence and burden of DENV, vector distribution, and viral serotypes and genotypes. Currently, there is no effective antiviral for dengue fever [42]. Proper implementation of routine diagnosis and management of early-diagnosed illnesses reduces morbidity and fatality rates. It would be interesting to explore differential diagnosis with other infectious diseases with close clinical presentations, such as malaria. There is also an issue of cross-reactivity of serological diagnostic techniques among arboviruses, including DENV [25, 42]. As a result, confirmatory tests such as real-time (quantitative) polymerase chain reaction (qPCR) and plaque reduction neutralization test (PRNT) are required to validate the findings of these serologic assays. Cost-effective control measures are required. Effective vaccine is available, but not currently available in Ethiopia. Because, treatment is supportive, vector management is the primary strategy for reducing the burden of DENV infection. The WHO integrated vector control strategy is useful in combating vector-borne diseases. This mechanism is environmentally safe when using insecticides for the elimination of mosquitoes’ larvae from their artificial and natural habitats [43].

This systematic review and meta-analysis estimates DENV seroprevalence in Ethiopia. We attempted to summarize the pooled prevalence of studies conducted in Ethiopia that reported the prevalence of DENV. However, this review has certain limitations. There is substantial heterogeneity between studies, *I*^2^ statistics may not be discriminative and should be interpreted with caution, avoiding arbitrary thresholds [44]. It may be due to diagnostic techniques, the time frame of sample collection, or the population. We did not conduct sub-group analysis because the studies have investigated different types of DENV markers of interest using different diagnostic techniques, time frames and populations. This review does not cover all parts of the country and studies were conducted in dengue fever suspected individuals and acute febrile patients. This may have an impact on the generalizability of our findings.

## Conclusion

The prevalence of DENV infection is became common among dengue fever suspected and febrile participants in Ethiopia. This suggests that healthcare providers, researchers and policymakers should give more attention to dengue fever. Significant levels of heterogeneity between studies were found, the result should be interpreted with caution. Routine and differential laboratory dengue virus diagnosis is required to detect cases early, manage cases appropriately and plan for outbreaks. Since, there is no effective treatment or available vaccine, prevention and vector control should be the primary DENV control mechanisms. Mosquito surveillance is essential for identifying mosquito breeding areas, and health education and environmental control can help to limit the spread of the dengue virus. To track DENV epidemiology in Ethiopia, a national arbovirus surveillance program should be implemented. Since studies included in this review is derived from dengue fever suspected and febrile patients, it may not represent nationwide prevalence. Studies should be conducted in communities and across the country.

### Electronic supplementary material

Below is the link to the electronic supplementary material.

Supplementary Material 1

Supplementary Material 2

Supplementary Material 3

Supplementary Material 4

## Data Availability

All data and materials are have included in the manuscript.

## Abbreviations

DENV: Dengue virus
DF: Dengue fever
DHF: Dengue Hemorrhagic Fever
DSS: Dengue Shock Syndrome
ELISA: Enzyme-linked immunosorbent assay
IFA: Immunofluorescence assay
IgG: Immunoglobulin G
IgM: Immunoglobulin M
RNA: Ribonucleic acid
RT: PCR-Real-time Polymerase Chain Reaction
WHO: World Health Organization
