# Supplementary material for: A dengue virus infection in Ethiopia: a systematic review and meta-analysis
Source: BMC Infect Dis. 2024 Mar 6;24:297. doi: 10.1186/s12879-024-09142-1 (PMC10918862; doi:10.1186/s12879-024-09142-1)
Supplement: Supplementary file 1 — Supplementary Material 1 [file 12879_2024_9142_MOESM1_ESM.docx]

**Search strategies for eligible articles**

1. **Pubmed search strategy**

(((((Dengue) AND (Ethiopia)) OR (("Dengue virus") AND (Ethiopia))) OR ((DENV) AND (Ethiopia))) OR (("Dengue Fever") AND (Ethiopia))) OR (("Dengue infection") AND (Ethiopia))

1. **Hinari search strategy**

(Dengue) AND ((Ethiopia) OR ("Dengue virus")) AND ((Ethiopia) OR (DENV)) AND ((Ethiopia) OR ("Dengue fever")) AND ((Ethiopia) OR (Dengue infection)) AND (Ethiopia)
