# Supplementary material for: A dengue virus infection in Ethiopia: a systematic review and meta-analysis
Source: BMC Infect Dis. 2024 Mar 6;24:297. doi: 10.1186/s12879-024-09142-1 (PMC10918862; doi:10.1186/s12879-024-09142-1)
Supplement: Supplementary file 3 — Supplementary Material 3 [file 12879_2024_9142_MOESM3_ESM.docx]

**Figure 6.** Funnel plot with 95% confidence limits of the pooled Immunoglobulin M marker of Dengue infection in Ethiopia
